# Similar or Different Effects? Quantifying the Effects of Humility and Modesty on Job Performance

**DOI:** 10.3389/fpsyg.2022.809841

**Published:** 2022-05-12

**Authors:** He Peng

**Affiliations:** School of Management, Fudan University, Shanghai, China

**Keywords:** modesty, humility, unethical pro-organizational behavior, innovative behavior, citizenship behavior

## Abstract

Humility and modesty are both emphasized in Eastern and Western societies. However, people usually conflate them in everyday usage. To reduce the confusion of the two constructs, it is very vital to carefully differentiate the two constructs and examine whether they lead to similar or different effects on job performance. In this study, we scrutinized the effects of the two constructs on four dimensions of job performance simultaneously, including task performance, citizenship behavior (helping and voicing), unethical pro-organizational behavior, and innovative behavior. Using a dataset of 239 employees and 77 supervisors, we showed that modesty is not related to task performance and voicing, but that it is positively related to unethical pro-organizational behavior and negatively related to helping and innovative behavior. In contrast, we showed that humility is negatively related to unethical pro-organizational behavior and positively related to task performance, helping, voicing, and innovative behavior. Our findings reveal that modesty and humility can lead to very divergent work outcomes. The results strongly support the idea that modesty and humility are distinct constructs embedded in separate nomological networks and strongly suggest that organizations should encourage employees’ humility rather than modesty. The theoretical and practical implications of this work are discussed.

## Introduction

Individual work role performance drives the success of organizations and even the entire economy (Morrison and Phelps, 1999; Campbell and Wiernik, 2015). Not surprisingly, job performance has been treated as the most crucial dependent variable in work psychology research (Campbell and Wiernik, 2015). Scholars have developed job performance as a multi-dimensional construct and investigated various factors that may predict it, including contextual factors such as leadership and organizational climate (e.g., Martin et al., 2016), and individual factors such as work experience (e.g., Quinones et al., 2001), cognitive ability (e.g., Schmidt and Hunter, 2004), emotional intelligence (e.g., O'Boyle et al., 2011), personality (e.g., Vinchur et al., 1998), and attitudes (e.g., Judge et al., 2001). To extend the literature, the present study aims to examine the effects of two important individual characteristics, i.e., humility and modesty, on employee performance.

As important positive character strengths and virtues, both humility and modesty have received increasing attention in organizational research recently because they are theoretically expected to bring organizations positive outcomes (Peterson and Seligman, 2004). Indeed, humility has been established as an important construct that can contribute to positive outcomes such as effective leadership (e.g., Collins, 2001; Vera and Rodriguez-Lopez, 2004; Owens and Hekman, 2012; Owens et al., 2013; Argandona, 2015). Modesty also has been validated as an important construct that may cause positive outcomes such as individuals’ likability and upward mobility (Tice et al., 1995; Gregg et al., 2008; McMullin, 2010; Blickle et al., 2012; Diekmann et al., 2015). Despite the progress made by scholars in research on humility and modesty separately, very few studies have empirically examined their relationship and compared the effects of the two constructs on work outcomes simultaneously.

However, humility and modesty are quite similar (Exline and Geyer, 2004; Gregg et al., 2008). Lay people usually conflate them together (Davis et al., 2016). For example, Gregg et al. (2008) identified the characteristics of being humble and non-boastful as main categories when they asked participants to describe their everyday understanding of modesty. Exline and Geyer (2004) also reported that when participants were asked to define humility, 44% used the word “modesty” in their definitions of humility or referred to modest behaviors, such as not bragging or not taking full credit for success. Weidman et al. (2018) reported similar results when they asked participants to describe humility. These qualitative studies all showed that lay people usually use modesty to define humility or use humility to define modesty. Even in academic research, some scholars have also not clearly differentiated the two constructs due to the overlap among them. Some scholars have used measures of modesty as proxies for humility (Rowatt et al., 2002) or have measured the two constructs together without differentiating between them (Lee and Ashton, 2004; Peterson and Seligman, 2004; Davis and Hook, 2014; Ou et al., 2014; Xiong et al., 2018). Thus, to reduce the confusion of the two constructs, it is very vital to carefully differentiate the two constructs and examine whether they lead to similar or different effects on job performance. As such, managers can better know how to manage employee humble and modest behavior and thus increase organizational performance.

Hence, we sought to answer the following question in this study: *Do humility and modesty lead to similar or different work outcomes*? To answer this question, we first reviewed and differentiated the two constructs. To more clearly and cleanly differentiate the two constructs, we define the two constructs from the interpersonal perspective because the overlap between modesty and humility mainly lies in the intrapersonal aspect. In the present study, humility (qianxu in Chinese) refers to interpersonal behavioral manifestations that reflects a person’s willingness to acknowledge personal limitations or mistakes, appreciate others’ strengths, and be open to feedback and advice from others (Owens et al., 2013). By contrast, modesty (didiao in Chinese) refers to a low-key or self-deprecating form of self-presentation (Xiong et al., 2018). The underlying motives of humility and modesty are different. Humble behavior is mainly motivated by the self-improvement motives (Owens et al., 2013; Argandona, 2015), which implies a very strong orientation for learning. Thus, humble behaviors mainly involve acts such as willingness to acknowledge mistakes and actively seek feedback that significantly related to performance improvement. By contrast, the modest behavior is mainly driven by the motives to protect oneself (Hu and Huang, 2009; Owens et al., 2013; Xiong et al., 2018). To prevent potentially negative consequences, individuals try to keep a low profile and even play down their positive traits, talents, and strengths (Cialdini and de Nicholas, 1989). It is reasonable to expect that the above differences between humility and modesty may lead to divergent effects on job performance.

Scholars have agreed that job performance is multidimensional and all sub-dimensions of performance are organizationally important (Viswesvaran and Ones, 2000; Rotundo and Sackett, 2002). Hence, to comprehensively understand the effects of humility and modesty, we examine four dimensions of job performance in the present study.

**Figure 1:**
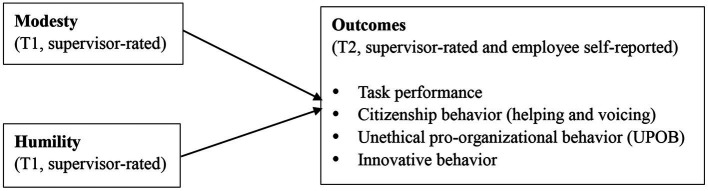
Research framework.

The first dimension is task performance, which is defined as individuals’ activities that may contribute to the core tasks of an organization (Borman and Motowidlo, 1993). Since humility entails a strong learning orientation that may facilitate task performance whereas modesty involves acts such as attention-avoiding and strengths-withholding that may impair individuals’ visibility, decrease received resources, and subsequently impair task performance, we expect humility is positively related to task performance, whereas modesty is negatively related to task performance. The second dimension is citizenship behavior, which refers to activities that may help organizations to pursue their goals by supporting the organizational, social, and psychological environment (Borman and Motowidlo, 1993). In line with the previous scholars’ recommendations (Van Dyne and Lepine, 1998), the present study focused on two forms of citizenship behavior: helping, which is affiliative, and voicing, which is challenging in nature. Helping refers to the act of consideration, whereas voicing refers to the expression of constructive suggestions for improvement (Van Dyne and Lepine, 1998). Since humility represents one aspect of reciprocal altruism (Trivers, 1971) that may facilitate citizenship behaviors whereas modesty entails intents such as attention-avoiding that may inhibit citizenship behaviors, we expect humility is positively related to helping and voicing, whereas modesty is negatively related to helping and voicing. The third behavioral dimension is unethical pro-organizational behavior (UPOB), which is defined as a set of unethical acts that seek to benefit the organization (Umphress et al., 2010; Mishra et al., 2021). Although UPOB is different from other performance-related constructs, scholars have shown increasing interest in this domain and have begun to investigate such behavior from a performance appraisal standpoint (Mishra et al., 2021). Since humility entails a self-transcendent pursuits that may make humble people to put public interest before organizations whereas modesty entails self-protection motives that may make modest people to put organization interest first (Hu and Huang, 2009; McMullin, 2010), we expect humility is negatively related to UPOB, whereas modesty is positively related to UPOB. Finally, given that scholars have recently called to include innovative behavior into the domain of job performance (Zhang et al., 2014), we also considered this aspect of performance in this study. Since humble people tend to be open for new information and then can boost innovation whereas modest people tend to avoid attention and then dare not to challenge the status quo, we expect that humility is positively related to innovative behavior, whereas modesty is negatively related to innovative behavior. Figure 1 outlines our research framework.

We contribute to the literature in several ways. First, by using multi-wave and multi-source data, we show that humility and modesty can predict very divergent work outcomes, providing strong evidence that the two constructs are distinct. Although previous scholars have discussed the differences between modesty and humility (Tangney, 2000; Owens et al., 2013), evidence for this distinction is mostly suggestive, and there is little empirical evidence to understand the difference in the predictive validity of these two constructs. Meagher et al. (2015) suggested that “the extent to which humility can be empirically disentangled from…modesty…, is still an open question” (p. 36). Davis et al. (2010) also expressed a similar idea that “[the researchers] have not differentiated it [i.e., humility] from some closely related constructs” (p. 243). Our research extend the literature by providing solid empirical evidence for the importance of differentiating between the two concepts in empirical or daily usage. Second, our research suggests that some good virtues may also encourage negative behaviors. Studies have shown the positive outcomes of modesty, such as greater career success and upward mobility (Blickle et al., 2012), likability (Diekmann et al., 2015), and positive investor reactions (Ridge and Ingram, 2017). However, we reveal that the modesty of employees may have a negative effect on organizations by inhibiting innovative behavior and facilitating UPOB. Thus, our study cautions that organizations should not have a naïve notion of employee modesty. More specifically, our results suggest that innovation-driven organizations should encourage their employees to exhibit humility rather than modesty.

## Theoretical Background and Hypothesis Development

### Humility, Modesty, and Their Distinction

#### Humility

Although humility has often mistakenly been described as low self-esteem, recently scholars have shifted to treat humility as a desired virtue and have thus begun to conceptualize it from a positive perspective (Tangney, 2000). Scholars have proposed several different definitions of humility from a trait or state perspective (Tangney, 2000; Ashton and Lee, 2005; Chancellor and Lyubomirsky, 2013). Most scholars have portrayed humility as a multi-dimensional construct. However, there is no agreement on the components of humility. For example, Tangney (2000) reviewed the philosophical, theological, and psychological literature and proposed six elements of humility: accurate assessment of one’s abilities and achievements; ability to acknowledge mistakes and imperfections; openness to new ideas; awareness of one’s abilities and accomplishments; recognition that one is but one part of the larger universe; and appreciation of the value of all things. Davis et al. (2010) proposed that relational humility should include four qualities: other-orientedness in one’s relationships with others; tendency to express positive other-oriented emotions; ability to regulate self-oriented emotions; and an accurate view of oneself. *Via* a series of qualitative and quantitative studies, Owens et al. (2013) further proposed that expressed humility includes three core components: admitting mistakes and limitations, appreciating others, and openness to feedback. Ou et al. (2014) suggested that in addition to the three dimensions specified by Owens et al. (2013), humility in leadership settings should include low self-focus, self-transcendent pursuit, and transcendent self-concept. Oc et al. (2015) proposed that the conception of leader humility in Singapore includes nine dimensions, such as leading by example and empathy and approachability.

Despite the ongoing debate over which qualities constitute the core of humility and which are simply related to humility, scholars have noted that the components of humility can be divided into intrapersonal and interpersonal aspects (Argandona, 2015). Intrapersonal aspects of humility mainly include self-knowledge, self-evaluation, and self-respect (Argandona, 2015). In contrast, interpersonal aspects of humility mainly include listening to others, seeking counsel and feedback, and allowing oneself to be known (Owens et al., 2013; Argandona, 2015).

#### Modesty

Although modesty is a potent and pervasive norm in Eastern culture, modesty has long been treated as an important virtue in Western culture (Ben-Ze’ev, 1993). Three main perspectives have been used to define modesty in the literature: trait perspective, intrapersonal perspective, and interpersonal perspective. Trait modesty refers to an individual’s dispositional tendency to be unassuming and self-effacing and to avoid bragging or expressing feelings of superiority. This trait has been treated as one facet of the agreeableness trait scale in NEO-PI-R and as one dimension of honesty-humility as a personality trait (Ashton and Lee, 2005).

Intrapersonal modesty refers to the set of modesty beliefs of an individual, including accurate self-view and consideration for others. Peterson and Seligman (2004) noted that “the term modesty refers primarily to the moderate estimation of one’s merits or achievements and also extends into other issues relating to propriety in dress and social behavior” (p. 463). Similarly, Sedikides et al. (2007) defined modesty as “a moderate self-view—viewing oneself as intermediate, rather than as very positive or very negative, on key personal attributes such as personality traits, abilities and skills, physical appearance, and social behavior” (p. 164). Consistent with this idea, Xiong et al. (2018) proposed a similar concept, value modesty, which refers to recognizing and persisting in the low-profile way of doing things as the ultimate goal. The definition of intrapersonal modesty is nearly identical to the definition of intrapersonal humility (Davis et al., 2016).

Interpersonal modesty refers to a set of behaviors that individuals try to withhold to avoid excessively presenting their personal strengths, such as special abilities, social relationships, accomplishments, honors, and contributions. From this viewpoint, modesty is a behavioral presentation style. For example, Wosinska et al. (1996) defined modesty as the “underpresentation of one’s positive traits, contributions, expectations or accomplishments” (p. 626). Similarly, Cialdini et al. (1998) defined modesty as “the public under-representation of one’s favorable traits and abilities” (p. 473). Blickle et al. (2012) followed this direction and operationalized modesty as an impression management tactic. Chen et al. (2009) also conceptualized modesty as a self-presentation tactic and examined the behavioral manifestations of modesty in different cultural contexts (especially in China and Canada). They identified three categories of modest behavior: self-effacement, enhancement of others, and attention-seeking avoidance. In the Chinese cultural context, some scholars have proposed the concept of instrumental modesty, which emphasizes that the aim of people’s modest behaviors is to realize firmness-restraining flexibility and strength-defeating weakness through unassuming self-presentation (Xiong et al., 2018). Instrumental modesty is situated and utilitarian, whereas value modesty is pan-situation and non-utilitarian (Xiong et al., 2018).

#### Distinction Between Humility and Modesty

From the above discussion, it is clear that both modesty and humility can be divided into intrapersonal and interpersonal components. To more clearly and cleanly differentiate the two constructs, we suggest that it is better to define the two constructs from the interpersonal perspective for two main reasons. First, the overlap between modesty and humility mainly lies in the intrapersonal aspect. Both intrapersonal modesty and intrapersonal humility involve an accurate or moderate self-view (Davis et al., 2016). It is difficult to differentiate humility from modesty using aspects of inner value. However, modesty and humility can be easily differentiated from behavioral manifestations. Second, the interpersonal perspective aligns with the other-reported rating of humility and modesty, which is more appropriate to measure the two constructs than self-report. Scholars have noted that self-report is inappropriate to assess the degree of humility and modesty (Tangney, 2005; Davis et al., 2011; Xiong et al., 2018), because people that are genuinely humble and modest will most likely not describe themselves as humble and modest (Richards, 1992; Comte-Sponville, 2001; Davis et al., 2011). Thus, other-report will be especially appropriate if humility and modesty are defined as behavioral constructs.

Therefore, in this study, we mainly followed the operational definition of humility of Owens et al. (2013) and the operational definition of modesty of Xiong et al. (2018) because they are both based on the behavioral approach. More specifically, humility (qianxu in Chinese) is defined as interpersonal behavioral manifestations that reflects a person’s willingness to acknowledge personal limitations or mistakes, appreciate others’ strengths, and be open to feedback and advice from others (Owens et al., 2013). Consistent with the analysis by Argandona (2015), the three components are the core unique behavioral features of humility. Humble behaviors essentially reflect open-mindedness, a willingness to admit mistakes and seek advice, and a desire to learn (Tangney, 2000). In contrast, modesty (didiao in Chinese) is defined as a low-key or self-deprecating form of self-presentation (Xiong et al., 2018). To get along with others, individuals may withhold or underrepresent their strengths or achievements (Cialdini and de Nicholas, 1989). Modest behavior reflects a desire to be accepted by others and a motive to protect oneself and create a good image (Owens et al., 2013; Xiong et al., 2018).

We suggest that there are two main differences between behavioral humility and behavioral modesty. First, the underlying purposes of humble behavior and modest behavior differ. As a presentation style, modest behavior is mainly driven by the motivation to protect oneself and to create a good image (Hu and Huang, 2009; Owens et al., 2013; Xiong et al., 2018). Individuals may engage in modest behaviors to create a good image and a positive impression of themselves in the eyes of others (Blickle et al., 2012). Alternatively, individuals may exhibit modest behaviors to protect themselves from the severe consequences of being envied (Chen et al., 2009; Hu and Huang, 2009; Xiong et al., 2018). To prevent potentially negative consequences, individuals try to keep a low profile, avoid drawing others’ attention to prevent them from being distinguished from other people, and even play down their positive traits, talents, and strengths (Cialdini and de Nicholas, 1989). In contrast, humility mainly stems from a sense of inner security (Exline and Geyer, 2004) rather than social approval. Humble behaviors are mainly driven by the motivation for self-improvement (Owens et al., 2013; Argandona, 2015), which implies a very strong orientation for learning.

Second, the behavioral manifestations of modesty and humility are very different. Humble behaviors mainly involve acts that intend to seek feedback or advice from others. In contrast, modest behaviors mainly involve keeping a low profile and possibly even withholding strengths or traits to avoid others’ attention. Humble behaviors reflect a person’s self-transcendent pursuits and moral principles (Nielsen et al., 2010), whereas modest behaviors mainly reflect self-interest (Xiong et al., 2018). Moreover, modest people tend to downplay or even intentionally withhold their strengths (e.g., achievements and close relationships with powerful people), whereas humble people are willing to let other people know them (Argandona, 2015). As Argandona (2015) noted, “he [a humble person] does not vaunt his strengths, but neither does he hide them…he shows an open, non-defensive attitude” (p. 65).

### The Effects of Humility and Modesty on Work Outcomes

As humility and modesty arise from different motives and have very different behavioral manifestations, we suggest that they should have divergent effects on work outcomes.

#### Task Performance

We posit that humility will boost individual task performance. There are several reasons for this. First, as humble people have a strong tendency to appreciate others’ strengths, they are more likely to notice the positive characteristics of higher performers and learn from them (Owens et al., 2013). Second, as humble people can recognize their own strengths and weaknesses, it is logical to speculate that they will be more likely to make suitable decisions at work (Owens et al., 2013). Third, as humility stems from a basic drive to improve oneself, humble people actively seek feedback from colleagues and leaders. Such feedback-seeking behavior subsequently improves their task performance (Lam et al., 2007). Finally, humble employees tend to be liked and encouraged by their leaders because of their other-oriented motivations (Morris et al., 2005); thus, they can more easily develop good relationships with their leaders and obtain more resources, such as feedback, support, and mentoring (Li et al., 2021). Subsequently, more resource support and better leader–member exchange will increase employees’ task performance. Empirically, scholars have identified a significant and positive correlation between employees’ humility and task performance (Owens et al., 2013; Li et al., 2021). Hence, it is reasonable to expect a positive relationship between humility and individual task performance.

In contrast, the relationship between modesty and employees’ task performance is more complex. On the one hand, employees’ modesty may increase task performance because a modest self-presentation may be expected by coworkers and supervisors and hence it may increase mentoring from supervisors (Blickle et al., 2008) and support from coworkers. On the other hand, as modesty entails the under-representation of a person’s traits, abilities, and achievements, modesty will hamper one’s visibility (Simpson and Lewis, 2005; Lewis and Simpson, 2012), which further decrease the resources received from bosses and subsequently impair ones’ task performance. Indeed, scholars have noted that invisibility may cause individuals to be marginalized or denied (Simpson and Lewis, 2005). Thus, compared to immodest people, modest people may receive less resources from their bosses. Subsequently, their task performance will be negatively affected.

Given the finding that the positive effects of a modest presentation on task performance only manifest in some conditions (Diekmann et al., 2015), and given the under-representation nature of modesty, we expect that the potential negative effects of modesty task performance will outweigh the potential positive effects. Thus, we propose the following hypothesis.

*Hypothesis 1:* Humility is positively related to task performance, whereas modesty is negatively related to task performance.

#### Citizenship Behavior

Citizenship behavior refers to acts that are “discretionary, not directly or explicitly recognized by the formal reward system and that in the aggregate promote the effective functioning of the organization” (Organ, 1988, p. 4). Although citizenship behavior includes a wide range of behaviors, we only focused on helping and voicing in this study by following the Van Dyne and LePine (1998). Helping is a type of affiliative promotive behavior, whereas voicing is an example of challenging behavior. As voicing involves making innovative suggestions for change, even if others disagree, it is risky for the voicer. Both helping and voicing are important for improving organizational effectiveness and efficiency in a dynamic environment.

We posit that humility is positively related to individual citizenship behavior. Humble people value both themselves and others. They appreciate others’ strengths and acknowledge their own limitations, and they are more likely to hold beliefs of egalitarianism rather than superiority or servility in their communication with others (Chancellor and Lyubomirsky, 2013). According to Ashton and Lee (2007), humility represents one aspect of reciprocal altruism (Trivers, 1971). People who exhibit a “holier-than-thou” attitude and arrogance are more likely to blame others and defend themselves. In contrast, humble people are more likely to offer more of their time and energy to help others (LaBouff et al., 2012). In addition, highly humble people tend to cooperate with others and contribute to teamwork, even when they might be exploited by others (Li et al., 2021). Indeed, studies of highly humble leaders have shown that, unlike less humble leaders, highly humble leaders are more likely to emphasize the growth of followers, act in the best interests of others, and consider others’ needs when they make decisions (Morris et al., 2005). Thus, humility should be positively related to helping behavior. Given that voicing is operationalized as a construct rooted in the desire to improve the organization rather than to gain personal interest and that humility reflects reciprocal altruism (Ashton and Lee, 2007), humility is also expected to be positively related to voicing.

According to Rioux and Penner (2001), individuals may engage in citizenship behavior with three motives: personal social values (i.e., to be helpful to others), organizational concern (i.e., to benefit the organization), and impression management (i.e., to obtain rewards, such as pay rises and promotions). From the above discussion, we suggest that humble people engage in citizenship behavior mainly for personal social values and concern for the organization.

However, the relationship between modesty and citizenship behavior may mainly be explained by the impression management perspective. The impression management perspective suggests that individuals may use citizenship behaviors to achieve their own goals when they believe that such behaviors can be instrumental in pursuing their goals (Bolino, 1999). From this perspective, instrumental motives may include promotion, pay rises, and other rewards. For example, Hui et al. (2000) found that those who regarded citizenship behaviors as having instrumental value for promotion engaged in such behaviors before promotion but decreased such behaviors after promotion. Studies have mainly discussed instrumental motives that may foster citizenship behavior because citizenship behavior is typically considered desirable behavior (Bolino, 1999). However, as Bolino (1999) noted, citizenship is not always viewed as desirable behavior. People exhibit modesty mainly to avoid receiving attention from others and to protect themselves. Thus, we expect that highly modest people may view helping as undesirable because such behavior may draw unnecessary attention to themselves and increase their exposure to others. Indeed, Snell et al. (2013) interviewed 63 Chinese employees from Hong Kong and found that one of the reasons that subordinates attributed undervaluation of contextual performance was because they felt the need to keep a low profile.

In addition, voicing is fundamentally risky: voicer may be perceived as troublemakers and even be criticized publicly. Thus, to avoid being criticized and the center of the controversy, highly modest people should be strongly motivated to assess the risks associated with voicing and then engage less voicing. Therefore, we expect that modesty will also be negatively related to voicing. Thus, we propose the following hypotheses.

*Hypothesis 2:* Humility is positively related to helping, whereas modesty is negatively related to helping.

*Hypothesis 3:* Humility is positively related to voicing, whereas modesty is negatively related to voicing.

#### Unethical Pro-organizational Behavior

UPOB refers to “actions that are intended to promote the effective functioning of the organization or its members (e.g., leaders) and violate core societal values, mores, laws, or standards of proper conduct” (Umphress and Bingham, 2011, p. 622). The definition of UPOB has two important components. First, such behavior is unethical because it violates hyper-norms or globally held ethical standards. However, it should be noted that such behavior may not violate organizational norms. Second, the intent of such behavior is to benefit the organization or the agents rather than oneself. Although UPOB is intended to help organizations initially, its final results may be destructive to organizations in the long term (Umphress and Bingham, 2011). Scholars have identified several antecedents for UPOB, such as organizational identification, leaders’ behavior, and organizational politics (Mishra et al., 2021).

In this study, we posit that humility and modesty may affect employees’ UPOB differently. According to the theory of moral self, individuals with strong moral identities are particularly sensitive and reactive to ethical issues compared with those with weak moral identities (Aquino and Reed, 2002; May et al., 2015). Indeed, empirical evidence has shown that employees with strong moral identities are less likely to engage in UPOB (May et al., 2015). Scholars have noted that humble people recognize something greater than themselves and respect moral laws, responsibilities for others, and the larger community (Tangney, 2000; Morris et al., 2005). Humble people seek goals that are less about themselves and more about the greater good and moral principles (Morris et al., 2005; Ou et al., 2014). Thus, humble people should have a stronger moral identity than people who are considered as arrogant. As UPOB is unethical from a social perspective, we expect that people who exhibit a greater degree of humility are less likely to engage in UPOB.

Instead, modest behaviors are mainly driven by a person’s desire to protect themselves (Hu and Huang, 2009; McMullin, 2010), rather than by self-transcendent pursuits. Research has revealed that employees who perceive a risk or threat in an organization may demonstrate UPOB to gain acceptance in the organization due to self-interest (Ghosh, 2017). As modesty is mainly driven by the motive of self-protection, we expect that people who exhibit a high degree of modesty are more likely to be sensitive to the risks and threats in an organization, and they are thus more likely to engage in UPOB to be perceived positively in the organization and to protect themselves. Thus, we propose the following hypothesis.

*Hypothesis 4:* Humility is negatively related to unethical pro-organizational behavior, whereas modesty is positively related to unethical pro-organizational behavior.

#### Innovative Behavior

Innovation has been especially emphasized in this knowledge-based era because of its central role in the long-term survival of organizations. Innovative behavior refers to “the intentional proposal and application of novel and improved ideas, processes, practices, and policies aimed at organizational effectiveness, business success, and long-term sustainability” (Kwon and Kim, 2020, p. 2). We posit that humility can boost individuals’ innovative behaviors in two ways. First, humility can boost innovation by making people more open-minded. Innovation requires people to be truly open-minded and not emotionally defensive. As scholars have noted, humility reflects a strong tendency to learn through others (Owens et al., 2013) and to weigh information in a non-defensive way (Argandona, 2015). Humility is especially relevant in contexts that “entail frequent interactions with people and where the content of the interactions is to exchange information, feedback, and criticism” (Owens et al., 2013, p. 1519). Hence, we expect that by fostering open-mindedness, humility can help people to learn new things, be more tolerant of opposing views, and be more open to experiences, and hence foster creativity and innovative behavior (Hess, 2014; Deffler et al., 2016).

Second, humility can boost innovation by making people more empathetic. Empathy, which refers to the ability to share the feelings of another, is especially necessary for customer-centric innovation (Hess, 2014). Humility is a prerequisite for empathy (Hess, 2014). Indeed, empirical research has shown that the characteristics of humility, such as respecting others’ viewpoints and adopting a non-defensive stance, may enable people to more easily experience empathy (Krumrei-Mancuso, 2017). Thus, we argue that humility will be positively related to innovative behavior.

In contrast, we posit that modesty will negatively correlate with innovative behavior. First, innovation means doing things differently and challenging the status quo (Bagley, 2014), which will put the innovator at the center of attention. As modest people try to avoid others’ attention and try not to be differentiated from others, we believe that they will be less likely to engage in innovative behavior. Second, as innovation means that there is no standard procedure to follow, failures are unavoidable. Modest people should be particularly sensitive to potential failures because failures will attract the attention of others, cause a loss of face, and reduce likability (Leung et al., 2014). Given that fear of failure inhibits individual innovative behavior (Gurteen, 1998; Leung et al., 2014), we reason that modest people will be less likely to engage in innovative behavior because of this fear of failure. Third, given that innovative behavior is risky, and given that it is defined as a construct rooted in the desire to benefit the organization rather than to gain personal interest, a self-interested person may be less likely to engage in such change-oriented behavior (Moon et al., 2008). Hence, it is logical to expect that modest people driven by the motive of self-protection will be less likely to engage in innovative behavior. Thus, we propose the following hypothesis.

*Hypothesis 5:* Humility is positively related to innovative behavior, whereas modesty is negatively related to innovative behavior.

## Materials and Methods

### Procedures

We first recruited 77 managers who opted to voluntarily participate in our survey. All the managers were former or current MBA students at an Eastern China university. We then asked them to randomly select a maximum of five subordinates who would like to voluntarily participate in our survey. After obtaining consent from the subordinates, the managers provided us with their subordinates’ names. We recruited 261 subordinates. We then created the questionnaire code for each subordinate and manager, which was used to match the manager–subordinate questionnaires. Each questionnaire was sent to the participants in an envelope and with a note reminding them to put the completed questionnaire in the envelope and seal it before returning. In addition, in every questionnaire, we clearly stated the confidentiality of the academic survey and that all the data would be calculated in aggregation. We also stated clearly the voluntary nature of the survey and reminded the participants that they could withdraw at any time.

To test our hypotheses, we collected job performance data from both the managers and the subordinates. The survey was conducted in two waves. In the first wave, we sent the managers a questionnaire to ask them to rate their subordinates’ humility and modesty. One month later, we sent the managers the second-wave questionnaire, in which we asked them to rate their subordinates’ task performance, helping, voicing, UPOB, and innovative behavior. Meanwhile, we also sent the subordinates a questionnaire, in which we asked them to rate their own task performance, helping, voicing, UPOB, and innovative behavior. Finally, we collected 239 manager–subordinate dyadic datasets. The final employee sample consisted of 62.8% men and 37.2% women. Eighty-nine percent of the subordinates had a college degree or above. The mean organizational tenure of the subordinates was 3.47 years (SD = 2.89). The proportion of subordinates aged <26 years, 26–45 years, and ≥ 46 years was 17.6%, 59%, and 23.4%, respectively. With regard to industries, about 36.4% participants worked in IT and software service industry, 28% worked in manufacturing industry, 19.2% worked in banking and financial service industry, 9.2% worked in advertising industry, and 7% worked in trading and other industries.

### Measures

The managers reported their subordinates’ humility, modesty, task performance, helping, voicing, UPOB, and innovative behavior. In addition, the subordinates reported their own task performance, helping, voicing, UPOB, and innovative behavior. All the questionnaires were administrated in Chinese. All the original English questionnaires were translated into Chinese using a standard back-translation procedure (Brislin, 1970).

#### Humility

Humility was assessed using the nine-item scale proposed by Owens et al. (2013). Sample items include “This person is open to the ideas of others” and “This person admits when they do not know how to do something.” The items had five Likert-type response options ranging from 1 (strongly disagree) to 5 (strongly agree). Cronbach’s alpha for this scale was 0.88.

#### Modesty

We measured modesty using the 3-item scale from Ou et al. (2014) because these items are consistent with our definition. The items are “This person avoids attention to himself/herself,” “This person keeps a low profile,” and “This person is not interested in obtaining fame for himself/herself.” A 5-point Likert scale ranging from 1 (strongly disagree) to 5 (strongly agree) was used. Cronbach’s alpha for this scale was 0.87.

#### Task Performance

Task performance was assessed using the six-item measure from Tsui et al. (1997). These six items focus on the quantity, quality, and efficiency of employees. The supervisors (employees) were asked to indicate the extent to which they agreed that the employee’s (their own) performance was higher than that of other peers in a similar job. A sample item is “The quality of work is much higher than average.” The Likert-type response scale ranged from 1 (strongly disagree) to 5 (strongly agree). Cronbach’s alpha was 0.90 for self-rated task performance and 0.93 for supervisor-rated task performance.

#### Helping

Helping was assessed using the 7-item measure by Van Dyne and LePine (1998). A sample item is “(This person/I) volunteer(s) to do things for this work group.” The items had five Likert-type response options ranging from 1 (strongly disagree) to 5 (strongly agree). Cronbach’s alpha was 0.93 for self-rated helping and 0.91 for supervisor-rated helping.

#### Voicing

Voicing was assessed using the 10-item measure from Liang et al. (2012). This scale includes two dimensions (i.e., promotive and prohibitive). A sample item for promotive voice is “raise suggestions to improve the unit’s working procedure.” A sample item for prohibitive voice is “dare to point out problems when they appear in the unit, even if that would hamper relationships with other colleagues.” The response scale ranged from 1 (strongly disagree) to 5 (strongly agree). Cronbach’s alpha was 0.95 for self-rated voicing and 0.93 for supervisor-rated voicing.

#### Unethical Pro-organizational Behavior

UPOB was assessed using the six-item measure proposed by Umphress et al. (2010). A sample item is “If it would help the organization, this person (I) would misrepresent the truth to make the organization look good.” The items had five Likert-type response options ranging from 1 (strongly disagree) to 5 (strongly agree). Cronbach’s alpha was 0.93 for self-rated UPOB and 0.91 for supervisor-rated UPOB.

#### Innovative Behavior

Innovation is usually defined as a process involving both the generation and implementation of ideas. Thus, we adopted the 6-item measure by Scott and Bruce (1994) to assess innovative behavior because this measure includes both the generation and implementation of ideas. Sample items include “Searches out new technologies, processes, techniques, and/or product ideas” and “Develops adequate plans and schedules for the implementation of new ideas.” The items had five Likert-type response options ranging from 1 (strongly disagree) to 5 (strongly agree). Cronbach’s alpha was 0.88 for self-rated innovative behavior and 0.92 for supervisor-rated innovative behavior.

#### Control Variables

Studies have shown that there are gender and age differences in modest self-presentation (Singh et al., 2002; Xiong et al., 2018). Hence, we included age and gender as controls. In addition, we included the level of education and length of organizational tenure as controls in our analysis.

### Analysis Strategy

To test our hypothesized model, we used Mplus 8.0 to conduct the statistical analysis. First, we conducted a series of confirmatory factor analyses (CFA) to confirm construct validity. Next, we conducted structural equational modeling (SEM) to test our hypotheses. As the supervisors were required to rate several subordinates’ job outcomes, we controlled for the potential nesting effect in all the CFA and SEM analyses.

## Results

### Construct Validity

To identify whether modesty is a distinct construct from humility, we first constrained the items for modesty and three dimensions of humility to load on their respective factors. We found that the four-factor model had an adequate fit: *X*^2^(48) = 105.63, *p* < 0.01, comparative fit index (CFI) = 0.94, root-mean-square error of approximation (RMSEA) = 0.07, standardized root-mean-square residual (SRMR) = 0.05. All the factor loadings ranged from 0.69 to 0.91. In addition, the three dimensions of humility were significantly correlated (*r* = 0.56–0.70, all *p* < 0.01). However, modesty was only related to the first dimension of humility (i.e., admitting personal limitations; *r* = 0.23, *p* < 0.01), but it was not related to appreciation of others’ strengths (*r* = −0.07, *p* > 0.10) or openness to feedback (*r* = 0.10, *p* > 0.10).

We then tested a second-order model with a general factor to predict modesty and three dimensions of humility. Table 1 shows the CFA results. Although the model still showed an adequate fit [*X*^2^(50) = 120.05, *p* < 0.01, CFI = 0.93, RMSEA = 0.08, SRMR = 0.07], it was significantly worse than the four-factor model (△*X*^2^ = 14.42, *p* < 0.01, △CFI = 0.01, △RMSEA = 0.01, △SRMR = 0.02). In addition, the results showed that the general factor was strongly related to admitting personal limitations (*γ* = 0.70, *p* < 0.01), appreciation of others’ strengths (*γ* = 0.81, *p* < 0.01), and openness to feedback (*γ* = 0.86, *p* < 0.01), but it was not related to modesty (*γ* = 0.08, *p* > 0.10), suggesting that modesty and humility were different constructs.

**Table 1:** Model comparison.

Models	*X* ^2^	*df*	*p*	CFI	RMSEA	SRMR	△*X*^2^
1. Four-factor model^a^	105.63	48	<0.01	0.94	0.07	0.05	
2. Second-order model^b^	120.05	50	<0.01	0.93	0.08	0.07	14.42^**^

a In the four-factor model, we treated modesty and three sub-dimensions of humility (i.e., acknowledgment of personal limitations, appreciation of others’ strengths, and openness to feedback) as four distinct factors.

b In the second-order model, we added a second factor for modesty and three dimensions for humility.

** *p* < 0.01.

Finally, we conducted a series of CFAs on the 12 studied variables to examine their discriminative validity. Too many items may decrease the sample-size-to-parameter ratio; however, parceling can help to alleviate this problem (Little et al., 2002; Sass and Smith, 2006). We randomly parceled three indicators for performance, helping, and UPOB, respectively. For voicing and humility, we used sub-dimensional scores as indicators. The results showed that the 12-factor model fitted the data very well [*X*^2^(461) = 693.83, *p* < 0.01, CFI = 0.95, RMSEA = 0.05, SRMR = 0.05], much better than any other alternative model, including the one-factor model [*X*^2^(50) = 4,142.67, *p* < 0.01, CFI = 0.26, RMSEA = 0.17, SRMR = 0.19]. Table 2 shows the CFA results for some typical alternative models and demonstrates that the 12-factor model was appropriate for data interpretation.

**Table 2:** Construct validity for the studied variables.

Models	*X* ^2^	*df*	*p*	CFI	RMSEA	SRMR	△*X*^2^
1. Twelve-factor model^a^	693.83	461	<0.01	0.95	0.05	0.05	-
2. Eleven-factor model^b^ (i.e., combining supervisor-reported helping and supervisor-reported voicing)	789.83	472	<0.01	0.94	0.05	0.05	96^**^
3. Ten-factor model^b^ (i.e., combining supervisor-reported helping and voicing; combining self-reported helping and voicing)	926.27	482	<0.01	0.91	0.06	0.06	232.44^**^
4. Nine-factor model^b^ (i.e., combining all self-reported and supervisor-reported helping and voicing items into one factor)	1,615.11	491	<0.01	0.77	0.10	0.14	921.28^**^
5. One-factor model (i.e., combining all items into one factor)	4,142.67	527	<0.01	0.26	0.17	0.19	3,488.84^**^

a In the 12-factor model, we treated modesty, humility, self-reported and supervisor-reported performance, helping, voicing, unethical pro-organizational behavior (UPOB), and innovative behavior as distinct factors.

b There are other alternative models, but we only show a typical model here for simplicity. In addition, we do not present alternative eight-factor, seven-factor, five-factor, four-factor, three-factor, or two-factor models for simplicity.

** *p* < 0.01.

### Common Method Bias Check

To alleviate common method bias, we collected data from multi-sources and in multi-waves. However, since supervisors reported both independent and dependent variables, we still conducted Harman’s single-factor test to evaluate the common method variance. Specifically, we conducted exploratory factor analysis (EFA) for all items from the supervisor-rated constructs. EFA indicated that 32.79% (less than 50% of the recommended threshold) of the total variation was extracted by one factor. Thus, there is no problem with common method bias in our study.

### Descriptive Statistics

Table 3 shows the correlations among the studied variables. Modesty was not related to task performance, helping, and voicing, but it was positively related to UPOB (self-rated: *r* = 0.14, *p* < 0.05; supervisor-rated: *r* = 0.28, *p* < 0.01) and negatively related to innovative behavior (self-rated: *r* = −0.13, *p* < 0.05; supervisor-rated: *r* = −0.15, *p* < 0.05). Humility was positively related to performance (self-rated: *r* = 0.18, *p* < 0.01; supervisor-rated: *r* = 0.46, *p* < 0.01), helping (self-rated: *r* = 0.19, *p* < 0.01; supervisor-rated: *r* = 0.60, *p* < 0.01), voicing (self-rated: *r* = 0.24, *p* < 0.01; supervisor-rated: *r* = 0.47, *p* < 0.01), and innovative behavior (self-rated: *r* = 0.15, *p* < 0.05; supervisor-rated: *r* = 0.33, *p* < 0.01), but it was negatively related to UPOB (supervisor-rated: *r* = −0.25, *p* < 0.01). These initial findings supported Hypotheses 4 and 5 and partial support for Hypotheses 1–3.

**Table 3:** Mean, SD, and correlation among the studied variables (*N* = 239).

	Mean	SD	1	2	3	4	5	6	7	8	9	10	11	12	13	14	15	16
1. Age	2.09	0.71	-															
2. Gender	0.63	0.48	−0.03	-														
3. Education	2.56	0.82	−0.09	−0.06	-													
4. Tenure	3.47	2.89	0.45^**^	−0.02	0.12^†^	-												
5. Modesty	3.09	0.82	−0.10	0.03	0.03	−0.11^†^	(0.87)											
6. Humility	3.51	0.58	0.10	−0.11^†^	0.03	0.18^**^	0.07	(0.88)										
7. Performance (self-rated)	3.67	0.82	0.16^*^	0.06	0.06	0.11^†^	−0.04	0.18^**^	(0.90)									
8. Helping (self-rated)	4.08	0.75	0.21^**^	0.07	−0.01	0.14^*^	−0.12^†^	0.19^**^	0.53^**^	(0.93)								
9. Voicing (self-rated)	3.76	0.82	0.25^**^	0.05	−0.15^*^	0.12^†^	−0.05	0.24^**^	0.52^**^	0.64^**^	(0.95)							
10. UPOB (self-rated)	2.41	1.15	−0.05	0.14^*^	−0.20^**^	−0.14^*^	0.14^*^	0.01	0.21^**^	0.10	0.13^*^	(0.93)						
11. IB (self-rated)	3.67	0.81	0.15^*^	0.04	−0.04	0.06	−0.13^*^	0.15^*^	0.57^**^	0.61^**^	0.61^**^	0.20^**^	(0.88)					
12. Performance (supervisor-rated)	3.39	0.83	0.08	−0.07	0.23^**^	0.24^**^	0.02	0.46^**^	0.25^**^	0.20^**^	0.19^**^	−0.05	0.13^*^	(0.93)				
13. Helping (supervisor-rated)	3.69	0.67	0.05	−0.06	0.13^*^	0.14^*^	0.04	0.60^**^	0.19^**^	0.15^*^	0.14^*^	0.08	0.09	0.57^**^	(0.91)			
14. Voicing (supervisor-rated)	3.25	0.73	0.09	−0.03	0.24^**^	0.29^**^	−0.08	0.47^**^	0.26^**^	0.18^**^	0.17^**^	−0.08	0.12^†^	0.65^**^	0.65^**^	(0.93)		
15. UPOB (supervisor-rated)	2.53	0.97	−0.13^*^	0.18^**^	−0.11	−0.11^†^	0.28^**^	−0.25^**^	−0.01	−0.07	−0.09	0.13^†^	−0.01	−0.02	−0.05	−0.07	(0.91)	
16. IB (supervisor-rated)	3.42	0.79	0.03	−0.06	0.11	0.08	−0.15^*^	0.33^**^	0.22^**^	0.16^*^	0.16^*^	−0.08	0.21^**^	0.64^**^	0.36^**^	0.42^**^	−0.04	(0.92)

Age: 1 = ≤25 years; 2 = 26–35 years; 3 = 36–45 years; 4 = 46–55 years; and 5 = > 55 years. Gender: 1 = male; 0 = female. Level of education: 1 = high school and below; 2 = college; 3 = Bachelor’s degree; 4 = Master’s degree; and 5 = doctoral degree. UPOB, unethical pro-organizational behavior and IB, innovative behavior. Alpha values are presented on the diagonal in brackets.

** *p* < 0.01;

* *p* < 0.05;

† *p* < 0.10.

### Hypothesis Testing

**Figure 2:**
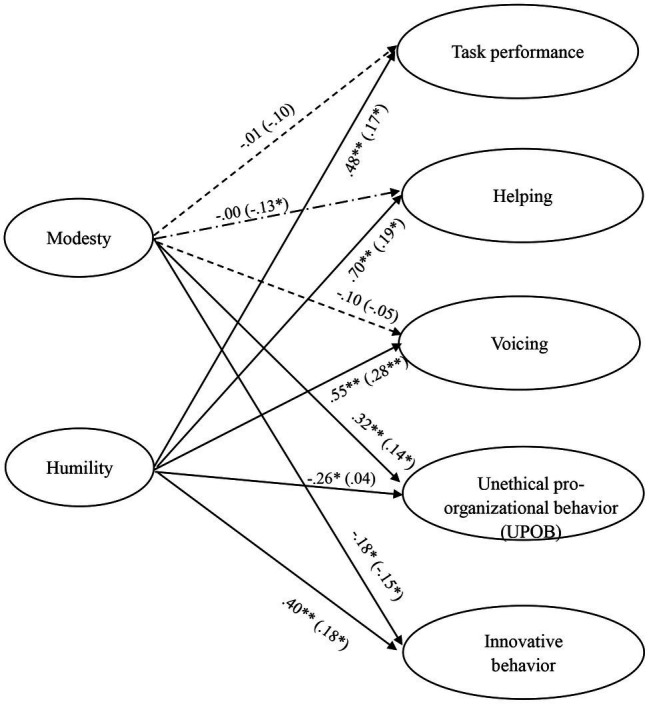
SEM Results for the hypothesized model. (1) The values outside the brackets are the path coefficients for the supervisor-rated outcomes; the values in brackets are the path coefficients for the employee self-rated outcomes. All the coefficients are standardized. (2) We controlled for age, gender, education, and organizational tenure for all outcome variables. For simplicity, we do not show the coefficients here. (3) ^**^p < 0.01 and ^*^p < 0.05.

To test our proposed hypotheses, we conducted SEM analyses using Mplus version 8.0. Our proposed model fitted the data very well (*X*^2^ = 887.13, *p* < 0.01, degrees of freedom [*df*] = 557, RMSEA = 0.05, CFI = 0.94, SRMR = 0.05). The results for the proposed paths are exhibited in Figure 2.

Hypothesis 1 states that humility is positively related to task performance, whereas modesty is negatively related to task performance. Our results revealed that humility was positively related to task performance (self-rated: *β* = 0.17, *p* < 0.05; supervisor-rated: *β* = 0.48, *p* < 0.01). However, modesty was not significantly related to task performance, although the coefficients were negative. Hence, Hypothesis 1 was only partially supported.

Hypothesis 2 states that humility is positively related to helping, whereas modesty is negatively related to helping. Our results showed that humility was positively related to helping (self-rated: *β* = 0.19, *p* < 0.05; supervisor-rated: *β* = 0.70, *p* < 0.01). Despite modesty was not significantly related to supervisor-rated helping, it was negatively related to employee self-reported helping (*β* = −0.13, *p* < 0.05). Hence, Hypothesis 2 was marginally supported.

Hypothesis 3 states that humility is positively related to voicing, whereas modesty is negatively related to voicing. Our results showed that humility was positively related to voicing (self-rated: *β* = 0.28, *p* < 0.01; supervisor-rated: *β* = 0.55, *p* < 0.01). However, modesty was not significantly related to voicing, although the coefficients were negative. Hence, Hypothesis 3 was only partially supported.

Hypothesis 4 states that humility is negatively related to UPOB, whereas modesty is positively related to UPOB. Our results showed that humility was negatively related to supervisor-rated UPOB (*β* = −0.26, *p* < 0.01), although it was not related to employee self-rated UPOB. In addition, modesty was positively related to UPOB (self-rated: *β* = 0.14, *p* < 0.05; supervisor-rated: *β* = 0.32, *p* < 0.01). Hence, Hypothesis 4 was marginally supported.

Hypothesis 5 states that humility is positively related to innovative behavior, whereas modesty is negatively related to innovative behavior. Our results showed that modesty was negatively related to innovative behavior (self-rated: *β* = −0.15, *p* < 0.05; supervisor-rated: *β* = −0.18, *p* < 0.05). In addition, humility was positively related to innovative behavior (self-rated: *β* = 0.18, *p* < 0.05; supervisor-rated: *β* = 0.40, *p* < 0.01). Thus, Hypothesis 5 was supported.

## Discussion

In this study, we empirically investigated the effects of humility and modesty on job performance simultaneously. Results showed that the effects of humility and modesty on employee job performance were divergent.

First, we examined the effects of humility and modesty on task performance. As expected, we found a positive relationship between humility and task performance, which is consistent with previous studies conducted in China (Li et al., 2021) and Germany (Wendler et al., 2018). For example, Li et al. (2021) found that employee humility could promote employees’ objective job performance *via* employee advice network centrality. However, unexpectedly, we found that modesty was not related to individual task performance. Diekmann et al. (2015) once employed a German sample and also found that there was no significant relationship between impression modesty and task performance. But they found that the relationship between impression modesty and task performance was moderated by trait modesty. This indicates that the relationship between modesty and job performance might be buffered by some factors. Future research could examine the potential moderators in the link between modesty and task performance.

Second, we examined the effects of humility and modesty on citizenship behaviors. As expected, we found that humility was positively related to helping and voicing, which is in line with previous studies in China (Shaw and Mao, 2021) and other countries (e.g., United States sample: LaBouff et al., 2012; South Korea sample: Oh et al., 2014; Germany sample: Wendler et al., 2018). For example, Shaw and Mao (2021) collected data from China and found that follower humility was positively related to follower voice. LaBouff et al. (2012) employed United States samples and found that humble people tended to help others. With regard to the relationship between modesty and citizenship behaviors, we unexpectedly found that modesty was not related to voicing. A potential explanation for this is that the relationships between modesty and voicing might be moderated by individual and situational factors. For example, studies have revealed that self-monitoring and political skills may influence the effects of behavioral modesty (Blickle et al., 2008, 2012). Future research could investigate the potential moderators in the link between modesty and voicing. In terms of the relationship between modesty and helping, we found that modesty was negatively related to self-rated helping, but not related to supervisor-rated helping. Since individuals tend to overstate their own desirable attributes and behaviors due to the social desirability bias, self-ratings on helping may be distorted. Hence, we should be cautious about the negative association between modesty and self-rated helping. Further research on the relationship between modesty and helping is needed.

Third, we examined the effects of humility and modesty on UPOB. As expected, we found modesty was positively related to both supervisor-rated and self-rated UPOB. However, in terms of the relationship between humility and UPOB, our results showed that humility was only negatively related to supervisor-rated UPOB, but not related to self-rated UPOB. Since previous scholars noted that self-rating might be more appropriate than other-rating for measuring UPOB (Umphress et al., 2010), we should be cautious about the negative association between humility and supervisor-rated UPOB. Moreover, although previous studies in western cultures mainly revealed that humility is negatively related to counterproductive work behavior (Italy sample: Chirumbolo, 2015; United States sample: Wiltshire et al., 2014) and unethical behavior (Germany sample: Pfattheicher and Bohm, 2018), a recent study conducted in Chinese culture found that humility even may positively lead to UPOB *via* moral credits (Darren et al., 2021). Hence, further research on the link between humility and UPOB is needed.

Fourth, as expected, we found that modesty was negatively related to supervisor-rated and self-rated innovative behavior, whereas humility was positively related to supervisor-rated and self-rated innovative behavior. To our knowledge, we are one of the first to examine the effects of individual humility and modesty on innovative behavior. Given that innovation has been one of the key factors to obtain organization success, our findings are especially vital. Our findings indicate that if organizations want to foster organization innovation, they should try to create a culture that can encourage employee humility. In contrast, managers should avoid building a culture of modesty that may encourage employee modesty.

## Theoretical Implications

This study contributes to the humility and modesty literature in several ways. First, we contribute to the literature by revealing that the two constructs of humility and modesty can predict different work outcomes and hence provide solid evidence that the two constructs are distinct. Lay people often conflate the two constructs (Exline and Geyer, 2004; Gregg et al., 2008). Even in academia, some scholars have used the two words interchangeably and conflated them in their measurements (Lee and Ashton, 2004; Peterson and Seligman, 2004; Davis and Hook, 2014; Xiong et al., 2018). Although several scholars have mentioned that the two constructs are distinct (Tangney, 2000; Owens et al., 2013), there is a paucity of empirical evidence. Scholars have called for research to further differentiate the two closely related constructs (Davis et al., 2010; Meagher et al., 2015). As a response, in this study, we examined the divergent effects of the two constructs on job performance simultaneously to provide solid empirical evidence for the importance of differentiating the two concepts in empirical research and daily usage. Our results caution that we cannot treat modesty simply as a subdomain of humility.

Second, we contribute to the modesty literature by revealing the potential negative effects of modesty. Studies have mainly focused on the positive effects of modesty. For example, scholars have found that behavioral modesty can increase individuals’ likability and perceived competence (Diekmann et al., 2015), as well as enabling greater career success and upward mobility (Blickle et al., 2012), higher performance ratings (Diekmann et al., 2015), and positive investor reactions (Ridge and Ingram, 2017). However, our study revealed that modesty is negatively related to helping and innovative behavior and positively related to UPOB. Given that innovative behavior is very important for organizations to retain their competitiveness in this era, and given that UPOB can exert negative effects on an organization in the long term, our results suggest that although modesty can help individuals to gain better social approval and better career development, it can have a harmful impact on an organization.

Third, we contribute to the management literature by focusing on the humility of employees. Previous management studies on humility have largely focused on the humility of leaders, which is embedded in the leadership literature. Scholars have shown that the humility of leaders can bring positive outcomes for the team and organizations (Owens et al., 2013); however, little is known about the humility of employees (Li et al., 2021). This is unfortunate as the employee humility is vital and has implications for management practices. Management scholars have called for research on employee humility (Owens et al., 2019). As a respond to this call, our study showed that employees’ humble behaviors can effectively predict employees’ task performance, citizenship behavior, innovative behavior, and UPOB. Our results provide solid empirical evidence for the importance of fostering employees’ humble behaviors.

Fourth, our research sheds light on the innovation literature by revealing a new obstacle to innovation (i.e., modesty) that is salient in the Chinese context but has been neglected in the previous studies. Studies on innovation have identified various barriers (Kwon and Kim, 2020). In this study, we revealed that people may not engage in innovative behavior if they want to keep a low profile. Our research suggests that defensive impression management might be an important barrier for organizational innovation. Given that Chinese cultural norms place particular emphasis on keeping a low profile, our study revealed that such a traditional cultural norm may impede organizational innovation. Our research partially echoes the suggestion by Cheng (1999) that traditional Chinese culture may be obstructive to innovation.

Finally, we contribute to the UPOB literature by revealing that modesty can foster UPOB, but humility can inhibit it. Studies on the antecedents of UPOB have mainly paid attention to situational factors, such as leadership styles, and have seldom focused on individual factors (Mishra et al., 2021). In addition, studies have often used the social identity perspective, social exchange perspective, and social learning theory to explain UPOB formation (Mishra et al., 2021). Our study extends the literature by suggesting a new perspective; specifically, people may engage in UPOB because they want to maintain a low profile.

## Managerial Implications

This study has several important managerial implications. First, our findings revealed that employees’ humility and modesty have divergent effects on job performance. More specifically, humility had a positive effect on task performance, citizenship behavior, and innovative behavior, and a negative effect on UPOB. In contrast, modesty had a negative effect on helping and innovative behavior and a positive effect on UPOB. Given that people usually conflate humility and modesty, our research shows that managers should differentiate between them because they have very different predictive effects.

Second, Chinese culture especially emphasizes modesty (Xiong et al., 2018). Studies have shown that modesty can increase individuals’ likability and enhance the probability of career success (Blickle et al., 2012). However, our results showed that it can hamper organizational innovation and increase UPOB. Thus, our study cautions that organizations should not have a naïve notion of employees’ modesty. Our results suggest that organizations that want to foster innovation should encourage humility rather than modesty at work. Given that modesty may decrease relationship conflict and foster interpersonal harmony, organizations should encourage employees to conduct themselves in a manner that is low profile but work in a manner that is high profile (i.e., to be low-key with people and high-key with things). Organizations can remove the situational cues that may encourage employees to work in a low profile. For example, as modest behavior often stems from the motivation of self-protection and happens in insecure environments, organizations may inhibit employees’ modest behaviors by building a psychologically safe and inclusive organizational climate.

## Limitations and Future Directions

This study has some limitations that should be noted. First, this study was conducted in the context of Chinese culture. In Western and Eastern cultures, people may have different norms and attitudes toward modesty and humility (Chen et al., 2009; Xiong et al., 2018); thus, our findings may not be generalizable. Although modesty and humility have been regarded as important virtues in both Western and Chinese cultures, contemporary Western people are more inclined to emphasize self-uniqueness, self-promotion, positive self-presentation, and personal achievement than contemporary Eastern people (Markus and Kitayama, 1991; Yokota, 2012; Xiong et al., 2018). Western people do not tend to play down their strengths or talents to avoid others’ envy and protect themselves. In contrast, East Asian cultural norms value harmonious relationships. Chinese people often use modesty to repress themselves to avoid offending others and to protect themselves from the negative consequence of being envied in the workplace (Hu and Huang, 2009). Cai et al. (2011) showed that the expression of modesty is self-enhancing in Chinese culture but not in American culture. In addition, Chinese culture seldom emphasizes openness to feedback, which is a core component of humility. Thus, contemporary Western culture puts more emphasis on humility than modesty, whereas Chinese culture puts more emphasis on modesty than humility. Hence, to generalize our findings, we strongly encourage future research to replicate our study using Western samples.

Second, we only investigated the direct effects of modesty and humility on job performance, and we did not directly investigate the underlying mechanism or the boundary conditions. We believe that there are various individual and situational factors that may influence the effects of modesty and humility on work outcomes. For example, future research could explore the potential moderating effects of political skills and different leadership styles.

Third, we could not rule out the effects of previous job performance as we did not measure the previous job performance in time 1. Future research could include more control variables to rule out other explanations. In addition, our design was still correlational despite we measured independent variables and dependent variable at two times. Future research could employ experiment method to examine our research model.

## Conclusion

Using multi-wave and multi-source data from China, we simultaneously investigated the effects of employees’ humility and modesty on job performance. Our results suggest that modesty is not a sub-domain of humility. Compared with humility, modesty has different effects on work outcomes. Our results reveal the importance of differentiating between humility and modesty. They also suggest that organizations should encourage employees’ humility rather than modesty. Our study emphasizes that organizations, especially Chinese organizations, should not have a naïve notion of employees’ modesty.

## Data Availability Statement

The original contributions presented in the study are included in the article/supplementary material, further inquiries can be directed to the corresponding author.

## Ethics Statement

The studies involving human participants were reviewed and approved by the Research Ethics Committee of Department of Psychology, Fudan University. The patients/participants provided their written informed consent to participate in this study.

## Author Contributions

HP conceptualized the study, collected the data, analyzed the data, drafted the manuscript, and approved the submitted version.

## Funding

This research was funded by the National Natural Science Foundation of China (grant no. 71772047).

## Conflict of Interest

The author declares that the research was conducted in the absence of any commercial or financial relationships that could be construed as a potential conflict of interest.

## Publisher’s Note

All claims expressed in this article are solely those of the authors and do not necessarily represent those of their affiliated organizations, or those of the publisher, the editors and the reviewers. Any product that may be evaluated in this article, or claim that may be made by its manufacturer, is not guaranteed or endorsed by the publisher.
